# Education and Physical Health Trajectories in Later Life: A Comparative Study

**DOI:** 10.1007/s13524-018-0674-7

**Published:** 2018-05-21

**Authors:** Liliya Leopold

**Affiliations:** Department of Sociology, University of Amsterdam, Nieuwe Achtergracht 166, 1018 WV Amsterdam, The Netherlands

**Keywords:** Education and health, Life course, International comparison

## Abstract

The cumulative (dis)advantage hypothesis states that health disparities between education groups increase with age. The present study examined this hypothesis in a comparative analysis of the United States, the United Kingdom, the Netherlands, and Sweden. These countries offer sharp contrasts in the social conditions that may intensify or inhibit processes of cumulative (dis)advantage. Using harmonized panel data from the HRS, ELSA, and SHARE, the study applied Poisson multilevel regression models to trace changes in the number of chronic conditions and functional limitations of people aged 50–76 (*N* = 16,887 individuals; 71,154 observations). The four countries showed a clear gradient in levels of physical health and in the extent to which health trajectories were shaped by education. Across all ages and cohorts, health problems were most prevalent in the United States, less prevalent in the United Kingdom and the Netherlands, and least prevalent in Sweden. A similar cross-national gradient was found for the size of health gaps between education groups and for the extent to which these gaps widened with age. Gaps were largest in the United States, smaller in the United Kingdom and in the Netherlands, and smallest in Sweden.

**Electronic supplementary material:**

The online version of this article (10.1007/s13524-018-0674-7) contains supplementary material, which is available to authorized users.

## Introduction

Research has consistently shown that health gaps between higher- and lower-educated people increase over the life course (Dupre 2007; Kim 2008; Kim and Durden 2007; Leopold 2016; Lynch 2003; Mirowsky and Ross 2008; Willson et al. 2007). This evidence supports the *cumulative (dis)advantage hypothesis*, which states that education is associated with health-related advantages and disadvantages that accumulate with age, enforcing a steady increase in health disparities (Ross and Wu 1996).

In recent years, tests of the cumulative (dis)advantage hypothesis have been refined by greater attention to the social conditions in which individual health trajectories unfold. In an influential line of research demonstrating the importance of sociohistorical context, cohort studies have indicated a “rising importance” (Mirowsky and Ross 2008) of the cumulative (dis)advantage process (Delaruelle et al. 2015; Goesling 2007; Kim 2008; Lynch 2003). According to these studies, newer cohorts are increasingly exposed to conditions that intensify the cumulative advantages and disadvantages that education brings, including rising inequality in economic returns to education, exposure to environmental stressors, health knowledge, and health behaviors (Goesling 2007).

Although these cohort studies have shown that health inequality strongly depends on the context in which individual life courses unfold, cross-national comparative studies of health trajectories remain scarce (Stolz et al. 2017). Longitudinal evidence on how education shapes health across the life course is largely limited to the U.S. context. This limitation is important given that other country contexts introduce variation in key institutional factors that target inequalities in health. Among these factors are education systems that are more or less inclusive or stratifying, employment policies that are more or less protective in terms of unemployment and exposure to adverse working conditions, public support after negative life events that is more or less generous, and social policy interventions that are more or less effective in targeting risky health behaviors (Beckfield et al. 2015).

Although comparative studies have examined health inequality in the United States and other developed societies (Avendano et al. 2009, 2010), these assessments were based on cross-sectional data that preclude the separation of age and cohort effects—a key requirement in the study of health trajectories (Lynch 2003). Recent research has added further single-country studies that offer longitudinal assessments of the cumulative (dis)advantage hypothesis (Chen et al. 2010; Leopold 2016), but this evidence remains limited in comparative perspective because the studies varied strongly in terms of sampling frames, observation periods, scope of panel data, measures of health, and estimation methods.

In view of these limitations, I designed the present study to provide a comparative longitudinal investigation of differences in health trajectories between education groups. I analyzed data from the United States, the United Kingdom, the Netherlands, and Sweden—four countries that offer sharp contrasts with regard to the social conditions shaping individual health trajectories. Among developed societies, the United States and Sweden can be placed at opposite poles in terms of institutional factors that promote or prevent disparities in health-related resources, resulting in larger or smaller inequalities in terms of social security, access to health care, working conditions, educational opportunity, economic means, risky health behaviors, and exposure to stress after adverse life events. The United Kingdom and the Netherlands can be placed between these two poles, allowing for a more nuanced comparative assessment.

The analysis was based on panel data from the Health and Retirement Study (HRS), the English Longitudinal Study of Ageing (ELSA), and the Survey of Health, Ageing and Retirement in Europe (SHARE). These surveys are ideally suited for longitudinal comparative research because the data are highly similar in terms of sampling frames, observation periods, cohort range, and measures of education and health. Given that the samples of all three surveys include only middle-aged and older people, my analysis focuses on health trajectories in later life.

## Background

The cumulative (dis)advantage hypothesis describes a process by which initial advantages or disadvantages associated with a structural position translate into a systematic divergence in life course resources, opportunities, and risks (Dannefer 1987; Ferraro and Shippee 2009; Ferraro et al. 2009; O’Rand 1996). Applied to education and health, the cumulative (dis)advantage hypothesis posits that education structures advantages and disadvantages in key determinants of health, producing widening health gaps between education groups as people age (Lynch 2003; Mirowsky and Ross 2008; Ross and Wu 1996).

Education reproduces and magnifies early advantages and disadvantages of social background and strongly determines income, occupational status, and wealth in later life (Kerckhoff 1995; Spring 1976). Depending on social background, children grow up in stable or unstable families, attend better or worse schools, earn more or less, reach higher or lower occupational positions, and experience more or less of the allostatic load of stress associated with economic hardship, unemployment, and other negative life events (McEwen 1998). Moreover, those who attain higher education increase their capacity of processing information and their sense of personal control (Mirowsky and Ross 2007)—skills that contribute to acquiring and maintaining a healthy lifestyle.

Important aspects of the process of cumulative (dis)advantage are education differences in the timing and duration of exposure to stress, hardship, and unhealthy lifestyles as well as the occurrence and consequences of negative life events. Smokers and nonsmokers, for example, are almost equally healthy in their 20s. In middle and later stages of the life course, differences in functional limitations and chronic conditions that are attributable to smoking gradually unfold. The same applies to other health-related factors that are structured along educational lines. Because it takes years until adverse working conditions, economic hardship, and exposure to stress take their toll on health (Shuey and Willson 2014), the cumulative (dis)advantage hypothesis posits that education gaps widen steadily with age.

### Cumulative (Dis)advantage in Comparative Perspective

Studies about cumulative (dis)advantage of education for health have focused mainly on the U.S. context. It is not clear whether this evidence can be generalized to other societies because countries differ strongly in terms of institutional factors that may influence the association between education and health over the life course (Beckfield et al. 2015). Even the most developed countries differ strongly regarding inequality of educational opportunity, redistributive policies, labor market regulations, social protection against negative life events, access to and quality of health care, and policies that target risky health behaviors.

As shown in Table 1, the United States, the United Kingdom, the Netherlands, and Sweden offer sharp contrasts with respect to institutional factors that may strengthen or weaken the relationship between education and key determinants of health over the life course (Avendano and Kawachi 2014; DiPrete 2002; Leopold 2016). First, education systems differ in the extent to which they reproduce advantages and disadvantages of social background and determine inequality in labor market outcomes. Important features of education systems are standardization, stratification, access to higher education, and vocational orientation (DiPrete et al. 2017; Pfeffer 2008). The relationship between social origin and destination is weaker in countries with high standardization, low stratification, and a low share of private costs for higher education. Moreover, the relationship between education and labor market outcomes (such as earnings and occupation) is weaker in countries with a lower degree of vocational orientation (DiPrete et al. 2017; Pfeffer 2008).

**Table 1:** Determinants of health in Sweden (SE), the Netherlands (NL), the United Kingdom (UK), and the United States (US)

Determinants of Health	Indicators for Cross-National Differences	SE		NL		UK		US	Source
Education	Standardization	High	=	High	>	Low	=	Low	Pfeffer (2008)
	Stratification (tracking age)	16	>	12	<	16	=	16	Braga et al. (2013)
	Private spending on tertiary education as % of total spending on education	10.5	<	29.7	<	42.7	<	63.7	OECD (2017a)
	Vocational orientation	Low	<	High	>	Low	=	Low	DiPrete et al. (2017)
Income	Ratio of earnings of lower-educated (ISCED 0–2) to average earnings in 2006 (%)	87	<	73	<	62	<	46	OECD (2017b)
Work	Strictness of employment protection regarding temporary contracts	1.44	>	0.94	>	0.38	>	0.25	OECD (2017c)
	Percentage (%) of employees working very long hours	1.1	>	0.44	<	12.83	>	11.69	OECD (2017d)
	Minimum number of paid vacation days (2012)	25	>	20	<	28	>	0	ILO (2012)
Sickness	Maximum length of paid sick leave	No max.	>	2 years	>	28 weeks	>	0	Rho et al. (2009)
	Financial support in paid sick leaves in % of earnings	80	>	70	>	20.3	>	0	Rho et al. (2009)
Unemployment	Maximum duration of unemployment compensation in months	18	<	24	<	6	=	6	Werner and Winkler (2004)
	Unemployment benefits as a % share of last net wage	80	>	70		1-time payment		50	Werner and Winkler (2004)
Health Care	Health insurance coverage (%)	100	>	97.9	<	100	>	85.5	OECD (2017e)
	Number of health care workers per 1,000 of a population in 2004	75.93	>	74.8	>	56.2	<	56.9	
Health Behaviors	Percentage-point differences in ever having smoked between lower and higher educated (those 50–65 years old in 2004)	10	>	8	=	8	<	16	Own calculations^a^
	Percentage-point differences in being obese (BMI ≥30) between lower- and higher-educated people (aged 50–65 years in 2004)	7	=	7	<	11	<	14	Own calculations^a^

^a^Author’s calculations based on the samples of 50- to 65-year-old nonimmigrants in 2004 in SHARE, HRS, and ELSA.

As Table 1 illustrates, the Swedish education system is designed to limit the reproduction of advantages and disadvantages related to social background and to weaken the link between education and labor market. In the United Kingdom, and especially in the United States, the education system more strongly connects social background to education attainment: the quality of education is less standardized, and access to higher education is more dependent on economic resources. In both countries, however, the link between education and the labor market is relatively loose, allowing for mobility after education has been completed (DiPrete 2002). The Dutch education system strongly connects social origin to social destination and allows for less social mobility after education has been completed: education certificates are essential for labor market success.

The influence of education systems may be weakened or strengthened by other policies that target inequality in various domains of life. Regarding one of the most important determinants of health—namely, material means—economic policy directly affects inequality via redistributive taxation and protection against poverty. As shown in Table 1, income inequality between education groups (measured in 2006) is smallest in Sweden and largest in the United States (Organisation for Economic Cooperation and Development (OECD) 2017b), with the Netherlands and the United Kingdom situated in between. These differences suggest that lower-educated people in Sweden are less likely to accumulate health disadvantages associated with economic hardship than lower-educated people in the Netherlands, the United Kingdom, and especially the United States. Moreover, in the 1990s—a period that was highly relevant for employment trajectories of the cohorts examined in the present study—Sweden ranked highest among these four countries on the OECD index for employment protection, followed by the Netherlands, the United Kingdom, and the United States.

Another health-relevant factor is work–life balance. Research has shown that long work hours are negatively associated with health (Johnson and Lipscomb 2006). As Table 1 shows, the fraction of workers who work more than 50 hours per week is more than 10 times higher in the United Kingdom and in the United States than in Sweden and in the Netherlands. Regarding the number of paid vacation days, the strongest contrast is between the United States and all other countries. Although British, Swedish, and Dutch employees of all occupational groups are entitled to at least 20 days of paid vacation, there is no such guarantee for U.S. workers.

The four countries also differ in their protection of individuals against negative life events. Table 1 illustrates these differences in terms of social policy associated with long-term sickness and unemployment. All four indicators show that compensation is most generous in Sweden and in the Netherlands, less generous in the United Kingdom, and least generous in the United States. Given that adverse circumstances such as sickness and unemployment are more common among lower-educated people, these policies may moderate the relationship between education and health over the life course.

Another important institutional factor is access to high-quality health care (van der Wel et al. 2011). The Swedish, Dutch, and the British welfare states provide universal coverage with health insurance, although the quality (measured in terms of health care workers per 1,000 people) is higher in Sweden and the Netherlands than in the United Kingdom. In the United States, the quality of medical care is similar to that in the United Kingdom, but coverage with health insurance is lower because health insurance is not mandatory and entitlement to universal health insurance is limited to older ages.

Finally, the four countries differ in the extent of social inequality in risky health behaviors. As Table 1 shows, educational disparities in smoking and obesity are present in all countries, but their magnitude is smallest in Sweden and in the Netherlands, larger in the United Kingdom, and largest in the United States. Given that educational disparities in health behaviors constitute one of the main factors highlighted by the cumulative (dis)advantage hypothesis, these differences again point to a cross-country gradient in the extent to which health gaps grow over the life course.

Taken together, the institutional factors summarized in Table 1 suggest that health risks, and social inequalities therein, are most comprehensively addressed in the Swedish context, closely followed by the Netherlands. Conversely, the mechanisms underlying the accumulation of advantages and disadvantages of education for health appear to be more influential in the United Kingdom and the most influential in the United States. Based on these considerations, I examined three guiding hypotheses about cross-country differences in health trajectories of older adults. Specifically, I expected a consistent gradient across the four countries (such that United States > United Kingdom > Netherlands > Sweden) in terms of (1) the prevalence of health problems, (2) the magnitude of health gaps between education groups, and (3) the extent to which these gaps increased with age*.*

### Previous Research

Many studies have examined the validity of the cumulative (dis)advantage hypothesis in research on education and health. Although the vast majority of studies have supported this hypothesis, it has long been contested on theoretical and empirical grounds. Doubts were raised particularly in studies that reported narrowing or persistent health gaps between education groups at older ages (Herd 2006; House et al. 2005). More recent research has shown that patterns of narrowing health gaps emerged as artifacts in cross-sectional studies that could not disentangle age and cohort effects (Lynch 2003; Mirowsky and Ross 2008). Persistent patterns at older ages are now widely regarded as a result of preceding divergence (Kim 2008; Mirowsky and Ross 2008; Ross and Mirowsky 2010; Willson et al. 2007).

Moreover, studies have shown that mortality and panel attrition are higher among lower-educated respondents (Lynch 2003; Noymer 2001). Selection has long been considered a source of bias but is today increasingly acknowledged as an outcome of the cumulative (dis)advantage process: those who have accumulated disadvantages are more likely to withdraw from surveys for health reasons or to die prematurely (Dupre 2007; Ferraro et al. 2009).

Based on these developments, the current state-of-the-art in research on the cumulative (dis)advantage hypothesis are studies that use longitudinal data to disentangle age and cohort effects and complement the analysis by tests for selective attrition and mortality (Rohwer 2016). Studies from the United States that meet both criteria have supported the cumulative (dis)advantage hypothesis (Dupre 2007; Kim 2008; Kim and Durden 2007). Furthermore, U.S. studies have shown that the divergent pattern associated with this hypothesis has intensified across cohorts (Goesling 2007; Mirowsky and Ross 2008; Willson et al. 2007).

Comparative research on health inequality, however, remains almost exclusively based on cross-sectional data. Jürges (2010) compared education differences in chronic conditions and physical limitations among the population aged 50 and older in the United States and in 11 European countries, finding that health inequality by education was largest in the United States, followed by the United Kingdom, and that the Netherlands and Sweden ranked among the least unequal countries. Avendano et al. (2009) examined the relationship between wealth and chronic conditions in the United States, the United Kingdom, and other European countries, including Sweden and the Netherlands. Their results showed better health and less inequality health in the latter group. In the United Kingdom and especially in the United States, health was worse, and health inequality was larger.

Despite their merits in exploring international differences in health inequality, these studies could not examine health trajectories over the life course. Examining the hypotheses guiding the present study required a comparative longitudinal design.

## Data and Method

### Samples

I combined harmonized longitudinal data from three surveys. For the United States, I used data from the Health and Retirement Study (HRS); for the United Kingdom, data from the English Longitudinal Study of Ageing (ELSA); and for the Netherlands and Sweden, data from the Survey of Health, Ageing and Retirement in Europe (SHARE). The SHARE and ELSA were developed with the aim of providing European data that are comparable to the HRS (Börsch-Supan et al. 2013; Chien et al. 2014). The sampling frames, frequency of panel waves, and measures of sociodemographic characteristics and health are highly comparable among the three data sets.

All surveys collected data from target samples of noninstitutionalized individuals aged 50 and older. Data were collected via telephone interviews in the HRS and face-to-face interviews in ELSA and SHARE. I used data from an observation period between 2004 and 2012 in the United States, between 2004 and 2013 in Sweden and the Netherlands, and between 2002 and 2012 in the United Kingdom.

I restricted all samples to individuals who (1) participated in the baseline year (2004 for HRS and SHARE, 2002 for ELSA; see Willson et al. 2007) and (2) were aged 50–65 at this initial observation. Some of the HRS respondents were recruited earlier because data collection had already started in 1992. In 2004, the HRS sample was refreshed to represent the U.S. population aged 50 years and older. Because of sampling design, the black population is overrepresented in the HRS data. The ELSA started in 2002, but the sample was not refreshed in 2004; thus, the ELSA sample of 2004 would not be representative because of panel attrition after the first wave. I therefore used the ELSA sample recruited in 2002. To ensure that the sample in each country was representative for the noninstitutionalized population older than 50 years in the year of the initial observation, I used cross-sectional individual weights for the 2004 wave of SHARE and HRS and from the 2002 wave of ELSA in the multivariate models. The weights were made available by the data providers.

I excluded immigrants from the analysis because their educational degrees are not equivalent to the educational degrees of respondents from the host countries. After all restrictions, I pooled the analytic samples selected from the three surveys, resulting in a total sample size of 16,887 individuals and 71,154 panel observations.

### Measures of Health

I used chronic conditions and functional limitations as measures of health. Chronic conditions include diseases such as high blood pressure, diabetes, stroke, and lung disease. For my purposes, the measure of chronic conditions had two important benefits. First, individuals tend to be at a higher or lower risk of being diagnosed with one or more of these conditions depending on their long-term lifestyles and exposure to various health stressors (Sturm 2002). Second, the measure does not rely on respondents’ self-assessments. Instead, identical survey questions in HRS, ELSA, and SHARE asked the respondents to report only those conditions diagnosed by a doctor.

A potential issue in cross-national comparisons of chronic conditions concerns differences in access to health care and in the degree of medicalization. Because lower-educated people in the United States face higher costs for seeing a doctor, they might receive fewer diagnoses. Moreover, in medicalized countries where it is more common to consult a doctor even without major problems (Conrad 2005), the prevalence of diagnoses—but not necessarily the actual prevalence—of diseases might be higher (Crimmins et al. 2010). Given these potential issues related to chronic conditions as a measure of health, I complemented the analysis by self-reported functional limitations as an additional measure of health. This measure is not affected by cross-national differences in access to health care and medicalization.

I assessed chronic conditions and functional limitations as additive indices taking values 0–4 for chronic conditions and 0–10 for functional limitations. Table 2 shows descriptive statistics for both indices as well as for each chronic condition and functional limitation on which these indices are based. The data in Table 2 indicate that the four countries differed strongly in average health levels. The number of chronic conditions in the United States was approximately twice as high as in Sweden and the Netherlands and was 30 % higher than in the United Kingdom. In terms of functional limitations, cross-national differences were even stronger, revealing a similar gradient across the four countries.

**Table 2:** Descriptive statistics

	Sweden				Netherlands				United Kingdom				United States			
	Mean	SD	Min.	Max.	Mean	SD	Min.	Max.	Mean	SD	Min.	Max.	Mean	SD	Min.	Max.
Age and Cohort																
Age	62	5.50	50	74	61	5.44	50	74	62	5.56	50	76	62	5.40	50	73
Year of birth	1946	4.37	1939	1954	1947	4.36	1939	1954	1945	4.36	1937	1952	1946	4.64	1939	1954
Cohort^a^	58	4.37	50	65	57	4.36	50	65	57	4.36	50	65	58	4.64	50	65
Education																
Lower	.43				.52				.60				.49			
Intermediate	.30				.24				.26				.26			
Higher	.26				.24				.15				.25			
Health Measures																
Number of chronic conditions	0.45	0.66	0	4	0.42	0.64	0	4	0.56	0.70	0	4	0.88	0.87	0	4
High blood pressure	.30				.26				.39				.53			
Diabetes	.09				.08				.08				.19			
Stroke	.03				.02				.03				.06			
Lung disease	.03				.06				.06				.09			
Number of Functional Limitations	0.83	1.58	0	10	0.87	1.70	0	10	1.64	2.39	0	10	2.17	2.50	0	10
Walk 100 meters	.03				.05				.09				.11			
Sit 2 hours	.08				.07				.13				.20			
Get up from a chair	.13				.13				.22				.37			
Climb several flights of stairs	.10				.13				.29				.37			
Climb one flight of stairs	.03				.07				.10				.13			
Stoop, kneel, crouch	.23				.17				.32				.41			
Extend arms above shoulders	.05				.06				.10				.14			
Push or pull a large object	.04				.06				.14				.21			
Lift, carry weight over 5 kg	.11				.13				.19				.18			
Pick up a small coin from table	.02				.02				.04				.06			
Characteristics of the Sample																
Male	.46				.46				.46				.42			
Black	––^b^				––^b^				.03				.21			
Died	.02				.01				.07				.08			
Dropout	.44				.50				.39				.20			
Survey year	2008	3.26	2004	2013	2008	3.26	2004	2013	2007	3.56	2002	2012	2008	2.83	2004	2012
Number of waves	3.42	1.47	1	5	3.26	1.61	1	5	4.42	1.91	1	6	4.38	1.15	1	5
Number of Individuals	1,551				1,669				5,831				7,836			
Number of Observations	5,298				5,443				28,828				34,322			

*Notes:* Data for Sweden and the Netherlands are from SHARE; data for the United Kingdom are from ELSA; and data for the United States are from HRS.

^a^Cohort is defined as age at first observation.

^b^Information on race was not available in Sweden and the Netherlands.

### Education

Drawing on information about a respondent’s highest level of education collected upon first observation, I measured education categorically as lower, intermediate, and higher education. Lower education included respondents with primary education, lower secondary education, and high school dropouts; intermediate education consisted of respondents holding upper secondary education or postsecondary vocational degrees; higher-educated respondents included those with a bachelor’s degree or higher levels of tertiary education.

**Fig. 1:**
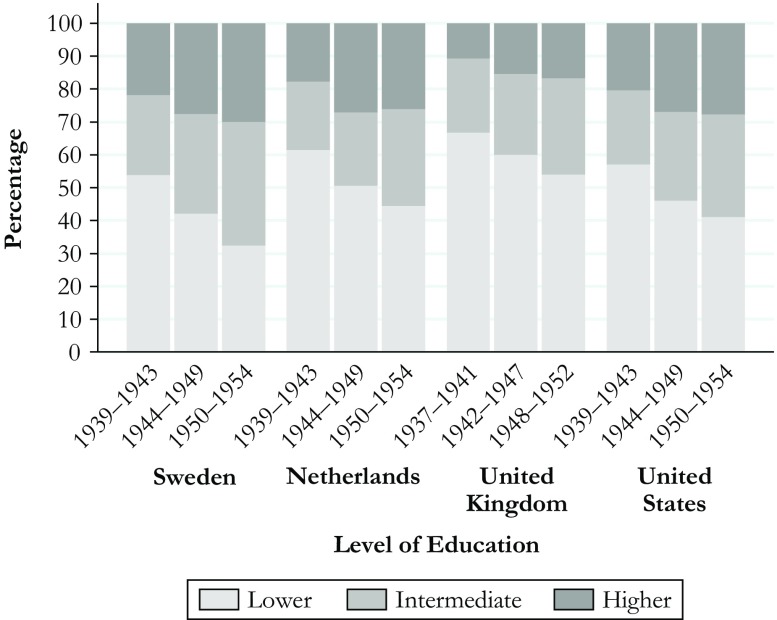
Distribution of education levels across cohorts. Data are from SHARE (Sweden and Netherlands), ELSA (United Kingdom), and HRS (United States)

Although these groups represented similar education categories in substantive terms, comparability regarding group sizes is important for interpreting results on the cumulative (dis)advantage hypothesis. A smaller group of lower-educated people may represent a more negative selection from the population. For example, if the group of lower-educated people would be smaller in the United States and the United Kingdom than in the Netherlands and Sweden, larger health gaps between education groups in the United States and the United Kingdom may result from selection rather than contextual differences. Moreover, the four countries differ in the scope and pace of educational expansion, resulting in cross-national variation in the extent to which the size of education groups changed across cohorts. Figure 1 offers a closer look at potential issues related to group sizes, plotting the distribution of education categories across cohorts in each country.

Figure 1 shows that the size of education groups is similar in Sweden, the Netherlands, and the United States. In the United Kingdom, the group of the lower-educated is larger, and the group of the higher-educated is smaller than in the United States, the Netherlands, and especially Sweden. This holds for each birth cohort. Although the cohorts in HRS, ELSA, and SHARE were conditioned on survival until the starting age (50–65) and on participation in the surveys, the data shown in Fig. 1 are consistent with data from the Cross-National Time-Series Data Archive (Banks and Wilson 2017) on international variation in the expansion of education.

### Age and Cohort

Age was measured in years, ranging from 50 to 73 in the United States, 50 to 74 in Sweden and the Netherlands, and 50 to 76 in the United Kingdom. Age was centered on the minimum of 50 years in the multivariate models. I added a quadratic age term (minimum-centered) to account for nonlinear trajectories. Birth years ranged from 1939 to 1954 in the United States, Sweden, and the Netherlands, and from 1937 to 1952 in the United Kingdom. I included birth cohort as a linear term representing age at first observation, centered on the minimum age of 50.

These parameterizations of age and cohort effects on the health outcomes were based on two criteria: (1) similarity between observed and fitted data examined by diagnostic plots, and (2) QIC (quasi-likelihood under the independence model).^1^ Inclusion of higher-level polynomials for age terms (cubic and quartic) and cohort terms (quadratic, cubic, and quartic), as well as interactions between education and higher-level polynomials of age and cohort, resulted in declines in similarity between observed and fitted data and in model fit compared with the preferred model in each country and in both health measures. This finding is in line with other studies on cumulative (dis)advantage based on similar data (Kim 2008; Mirowsky and Ross 2008).

### Analytic Strategy

I used multilevel, Poisson population-averaged regression models for count data (also known as GEE population-averaged models) to estimate change in the number of chronic conditions and functional limitations (Liang and Zeger 1986).^2^ The data included up to five observations per person in the United States (2004, 2006, 2008, 2010, and 2012), up to six observations per person in the United Kingdom (2002, 2004, 2006, 2008, 2010, and 2012), and up to four observations per person in Sweden and in the Netherlands (2004, 2007, 2011, and 2013). These repeated observations (Level 1) were nested within persons (Level 2). The multilevel estimation accounts for heterogeneity in health trajectories, allowing individual trajectories to differ in their starting levels and rates of change. According to the QIC criterion, an unstructured working correlation structure provided the best fit to the data.

Previous research has shown that models for change in the relationship between education and health should capture change with age, change across cohorts, and their interactions (Lynch 2003; Mirowsky and Ross 2008; Willson et al. 2007). To account for this, I included measures for age, cohort, and education as well as two- and three-way interactions between these measures. To examine cross-country differences, I interacted all variables with indicator variables for countries.

**Fig. 2:**
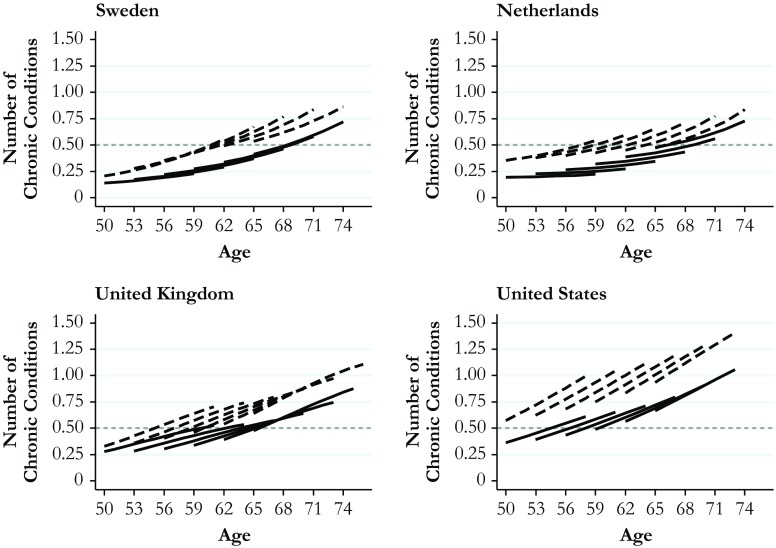
Predicted aging vectors of chronic conditions. Data are from SHARE (Sweden and Netherlands), ELSA (United Kingdom), and HRS (United States). Predictions are based on Model 1, Table S1 (Online Resource 1). Solid lines = Higher education; dashed lines = Lower education

**Fig. 3:**
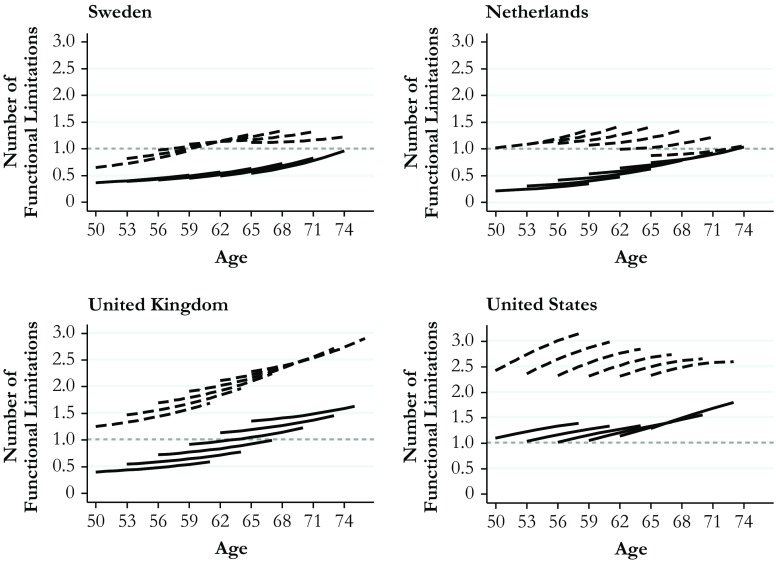
Predicted aging vectors of functional limitations. Data are from SHARE (Sweden and Netherlands), ELSA (United Kingdom), and HRS (United States). Predictions are based on Model 2, Table S1 (Online Resource 1). Solid lines = Higher education; dashed lines = Lower education

The results from the multivariate models are shown in Table S1 in Online Resource 1. In the presence of twofold, threefold, and fourfold interaction effects, the coefficients in Table S1 cannot be interpreted straightforwardly because they show estimates only for one specific combination of values. For example, the effect of cohort pertains to higher-educated Swedes at the minimum age. To facilitate the interpretation, I illustrate the results as age-vector graphs (Mirowsky and Kim 2007), which show age-related changes in average health levels among lower- and higher-educated people for different birth cohorts in each country. In upcoming Figs. 2 and 3, I present the main results for the number of chronic conditions and the number of functional limitations. To examine these results in more detail and to assess variability in the estimates, upcoming Tables 3 and 4 show the corresponding marginal differences in education along with their confidence intervals at the age of initial observation and 10 years later separately for six birth cohorts in each country. The marginal effects and their confidence intervals were calculated from Models 1 and 2 shown in Table S1.

**Table 3:** Estimated 10-year change in education differences in chronic conditions, by cohort

	Sweden		Netherlands		United Kingdom		United States	
Age at First Wave (birth cohort)^a^	Initial Difference	Change Over 10 Years	Initial Difference	Change Over 10 Years	Initial Difference	Change Over 10 Years	Initial Difference	Change Over 10 Years
50	0.0720	+0.184	0.205	+0.136	0.0457	+0.113	0.141	+0.279
(1954)	[–0.0449, 0.189]	[0.146, 0.221]	[0.0437, 0.366]	[0.125, 0.146]	[–0.0403, 0.132]	[0.092, 0.132]	[–0.0948, 0.377]	[0.101, 0.302]
53	0.104	+0.208	0.171	+0.152	0.0753	+0.113	0.186	+0.287
(1951)	[0.013, 0.195]	[0.147, 0.268]	[0.0690, 0.273]	[0.117, 0.187]	[0.0203, 0.130]	[0.093, 0.133]	[0.0295, 0.343]	[0.262, 0.313]
56	0.133	+0.214	0.134	+0.169	0.103	+0.109	0.223	+0.265
(1948)	[0.0593, 0.206]	[0.129, 0.293]	[0.0618, 0.206]	[0.106, 0.231]	[0.0609, 0.146]	[0.087, 0.129]	[0.125, 0.322]	[0.212, 0.317]
59	0.150	+0.188	0.0931	+0.187	0.130	+0.095	0.250	+0.225
(1945)	[0.0680, 0.233]	[0.124, 0.251]	[0.0129, 0.173]	[0.132, 0.243]	[0.0776, 0.182]	[0.0804, 0.111]	[0.177, 0.322]	[0.175, 0.277]
62	0.147	+0.131	0.0490	+0.209	0.154	+0.071	0.261	+0.165
(1942)	[0.0393, 0.254]	[0.048, 0.214]	[–0.0638, 0.162]	[0.125, 0.292]	[0.0788, 0.228]	[0.050, 0.093]	[0.178, 0.345]	[0.135, 0.195]
65	0.112	+0.043	0.00188	+0.234	0.172	+0.031	0.250	+0.077
(1939)	[–0.0373, 0.262]	[–0.213, 0.299]	[–0.169, 0.173]	[0.009, 0.46]	[0.0652, 0.279]	[–0.033, 0.094]	[0.132, 0.368]	[–0.030, 0.184]

*Notes:* Data are from SHARE (Sweden and Netherlands), ELSA (United Kingdom), and HRS (United States). Estimates are average marginal differences between lower- and higher-educated people in the number of chronic conditions; 95 % confidence intervals are shown in brackets. Estimates are based on Model 1, Table S1 (Online Resource 1). Initial differences are predicted mean differences in the number of chronic conditions at the age of first observation. Change over 10 years is calculated as a difference between predicted mean differences at initial observation and predicted mean differences 10 years later.

^a^In the United Kingdom, initial ages of 50, 53, 56, 59, 62, and 65 correspond to birth cohorts 1952, 1949, 1946, 1943, 1940, and 1937, respectively.

**Table 4:** Estimated 10-year change in education differences in functional limitations, by cohort

	Sweden		Netherlands		United Kingdom		United States	
Age at First Wave (birth cohort)	Initial Difference	Change Over 10 Years	Initial Difference	Change Over 10 Years	Initial Difference	Change Over 10 Years	Initial Difference	Change Over 10 Years
50	0.259	+0.266	0.821	+0.21	0.812	+0.225	1.347	+0.484
(1954)	[–0.00403, 0.523]	[0.137, 0.394]	[0.504, 1.137]	[0.121, 0.299]	[0.589, 1.035]	[0.221, 0.229]	[0.626, 2.067]	[0.448, 0.521]
53	0.404	+0.245	0.787	+0.147	0.897	+0.227	1.344	+0.324
(1951)	[0.197, 0.611]	[0.044, 0.446]	[0.573, 1.002]	[–0.004, 0.296]	[0.726, 1.068]	[0.196, 0.257]	[0.914, 1.774]	[0.255, 0.393]
56	0.539	+0.158	0.691	+0.071	0.964	+0.22	1.316	+0.167
(1948)	[0.338, 0.740]	[–0.035, 0.351]	[0.499, 0.884]	[–0.07, 0.211]	[0.821, 1.106]	[0.176, 0.264]	[1.059, 1.573]	[0.040, 0.294]
59	0.630	+0.017	0.538	–0.006	1.005	+0.205	1.264	+0.004
(1945)	[0.390, 0.870]	[–0.077, 0.111]	[0.312, 0.763]	[–0.095, 0.121]	[0.835, 1.174]	[0.181, 0.230]	[1.077, 1.451]	[–0.094, 0.102]
62	0.648	–0.144	0.343	–0.07	1.013	+0.185	1.182	–0.176
(1942)	[0.373, 0.923]	[–0.221, –0.068]	[0.0602, 0.625]	[–0.225, 0.085]	[0.765, 1.261]	[0.150, 0.220]	[0.982, 1.382]	[–0.231, –0.121]
65	0.581	–0.286	0.130	–0.113	0.985	+0.159	1.056	–0.388
(1939)	[0.254, 0.908]	[–0.512, –0.060]	[–0.248, 0.509]	[–0.491, 0.263]	[0.616, 1.355]	[0.011, 0.306]	[0.769, 1.343]	[–0.580, –0.196]

*Notes:* Data are from SHARE (Sweden and Netherlands), ELSA (United Kingdom), and HRS (United States). Estimates are average marginal differences between lower- and higher-educated people in the number of functional limitations; 95 % confidence intervals are shown in brackets. Estimates are based on Model 2, Table S1 (Online Resource 1). Initial differences are predicted mean differences in the number of functional limitations at the age of first observation. Change over 10 years is calculated as a difference between predicted mean differences at initial observation and predicted mean differences 10 years later.

^a^In the United Kingdom, initial ages of 50, 53, 56, 59, 62, and 65 correspond to birth cohorts 1952, 1949, 1946, 1943, 1940, and 1937, respectively.

## Results

Figures 2 and 3 show model-based predictions for initial health levels and for change in health among the higher-educated (solid lines) and the lower-educated (dashed lines). The left upper panel shows the results for Sweden, the right upper panel shows the results for the Netherlands, the left bottom panel shows the results for the United Kingdom, and the right bottom panel shows the results for the United States. To account for cohort effects, I fixed the variable for cohort at six values of age at initial observation, corresponding to the birth years of 1939, 1942, 1945, 1948, 1951, and 1954.^3^ The *y*-axis indicates the predicted average number of chronic conditions (Fig. 2) and the predicted average number of functional limitations (Fig. 3). In both figures, higher values indicate worse health. The scales are identical across countries, and a grey dashed reference line is included to ease the comparison of effect sizes.

The *x*-axis shows each cohort’s age at the beginning and at the end of the observation period. The youngest cohort, for example, was observed from age 50 until approximately age 60. An important advantage of the graphical age-vector analysis is that it shows not only education gaps in age trajectories of health but also cohort differences in these trajectories as well as the associated levels of health (Mirowsky and Kim 2007). Cohort effects are visible at the age overlaps across cohorts. If cohort effects are small, the cohort-specific curves connect; if cohort effects are larger, the pattern appears increasingly ragged.

The findings illustrated in Figs. 2 and 3, and the associated Tables 3 and 4, provide answers to the three hypotheses guiding the present study. First, with regard to average levels of health, I found the expected cross-national gradient using both measures of health. In the United States, the number of chronic conditions and functional limitations was higher than in the United Kingdom and much higher than in the Netherlands and Sweden. This cross-national gradient was most pronounced for functional limitations: it applied to higher- and lower-educated people in all cohorts and across the age range covered. A comparison between the United States and Sweden illustrates the magnitude of cross-national differences: the dashed curves pertaining to lower-educated Swedes remained below all corresponding curves for the United States across the range of ages and cohorts. Thus, lower-educated Swedes were in better health than both lower-educated and higher-educated Americans. Health levels of higher-educated Americans were comparable to those of lower-educated people in the United Kingdom and the Netherlands. The estimated number of chronic conditions and functional limitations in lower-educated Americans vastly exceeded those for all other groups.

Second, with regard to the magnitude of health gaps between education groups, the results show the smallest gaps in Sweden, followed by the Netherlands, the United Kingdom, and the United States. This cross-national gradient of health inequality in later life was most pronounced for functional limitations (Fig. 3) but also visible for chronic conditions (Fig. 2). Beyond this general pattern, the age-vector graphs indicate cross-national differences in terms of cohort effects. In the United States, the United Kingdom, and the Netherlands, most of the curves show a ragged cohort pattern, whereas the curves tend to connect in Sweden. Cohort effects were most pronounced among lower-educated Americans. In this group, the number of chronic conditions and functional limitations grew rapidly across cohorts observed at the same ages. As a result, the magnitude of health gaps increased across cohorts. In the United Kingdom, cohort effects did not differ appreciably between both education groups for either measure of health, resulting in health gaps that remained stable across cohorts. In the Netherlands, higher-educated people showed health improvements across cohorts, whereas lower-educated people showed health declines across cohorts. These diverging trends applied to both measures of health, resulting in a cross-cohort trend of growing health gaps between education groups.

Third, with regard to cumulative (dis)advantage over the later life course, Figs. 2 and 3 indicate the extent to which health gaps between education groups increased with age. Health gaps at initial observation were largest in the United States and smallest in Sweden, with the United Kingdom and the Netherlands positioned in between. These cross-national differences were more pronounced in functional limitations than in chronic conditions and applied for most birth cohorts. For example, among people born around 1950,^4^ education differences in the number of functional limitations at the initial observation at age 53 amounted to only 0.4 in Sweden. These differences were twice as large in the Netherlands, 2.5 times larger in the United Kingdom, and 3.5 times larger in the United States (see Table 4). As this cohort moved through their 50s, the gaps increased to 0.65 in Sweden, 0.91 in the Netherlands, 1.1 in the United Kingdom, and 1.7 in the United States. However, in Sweden, the Netherlands, and the United States, this divergent pattern in functional limitations emerged only in more recent cohorts and within the age range between the early 50s and the late 60s, after which the observation windows for younger cohorts were censored. In the United Kingdom, education differences in the number of functional limitations increased in each cohort and across the entire age range 50–76. For the other health measure—number of chronic conditions—results showed an age-related increase in education gaps for each country and each cohort (see Table 3), consistent with the pattern postulated by the cumulative (dis)advantages hypothesis.

Taken together, the findings for most of the cohorts and for both health measures showed a clear gradient across the four countries in the size of health gaps at initial and final observation. Gaps were largest in the United States, followed by the United Kingdom, the Netherlands, and Sweden. Differences between the United States and all three European countries were large, whereas differences between the European countries were smaller.

## Additional Analyses

Although the general pattern of findings supported my expectations, it was important to examine a number of factors that could influence comparative conclusions about health inequality. I conducted four additional analyses to assess the role of (1) gender differences, (2) racial differences, (3) regional differences, and (4) selective attrition.

### Gender Differences

Previous research has shown gender differences in age trajectories of health. U.S. studies have found the life course increase in health gaps between education groups to be stronger among women (Pudrovska 2014; Ross and Mirowsky 2010). To examine gender differences, I estimated the models separately for men and women. The results are shown in Figs. S1 and S2 in Online Resource 1. Health gaps between education groups differed by gender in each country. However, the gender-specific analyses showed that the general conclusions regarding country differences applied to both men and women.

### Racial Differences

Research from the United States has shown that blacks are in much worse health than whites at each level of education (Brown et al. 2016). Because black respondents constitute a substantial share of the U.S. sample, I estimated the models separately for blacks and whites in the United States. As illustrated in the bottom-left graph in Online Resource 1, Figs. S1 and S2, blacks were in worse health than whites at both education levels. However, each of the conclusions regarding my guiding hypothesis applied also when I compared only white Americans with the other country samples.

### Regional Differences

Recent research has demonstrated that health levels and the size of education gaps in health vary across states within the United States (Montez et al. 2017). To examine whether my results for the United States were driven mainly by specific regions, I conducted separate analyses for four regions in the United States (Northeast, Midwest, South, and West). The findings from these analyses, presented in Figs. S3 and S4 in Online Resource 1, showed that despite regional differences within the United States, all comparative conclusions of the present study applied to each of the four regions.

### Selective Attrition

Finally, I examined the possibility that countries differed regarding nonrandom attrition due to health problems or death. As shown in Table 2, the percentages of dropouts in the SHARE and ELSA samples were more than twice as the large as percentage of dropouts in the HRS sample. If dropouts are related to education and health, differential attrition may bias the comparative results.

To assess the role of selective attrition, I examined (1) the dropout risk among the lower-educated, (2) initial health levels among lower-educated dropouts compared with those who stayed in the panel, and (3) inverse probability weighting as a correction for potential bias due to attrition.

Table 5 shows three columns separately by country and level of education (higher or lower). Leftmost columns (labeled “Stayed”) pertain to those who remained under observation from the initial wave until the last wave, middle columns (“Dropped out”) pertain to those who left the panel before the last wave, and rightmost columns (“Died”) pertain to those who died before the last wave. The descriptive statistics show (1) how the prevalence of attrition differed by country and education, and (2) how health at initial observation was associated with subsequent attrition from the panel.

**Table 5:** Attrition analysis

	Sweden						Netherlands					
	Lower-Educated			Higher-Educated			Lower-Educated			Higher-Educated		
	Stayed	Left	Died	Stayed	Left	Died	Stayed	Left	Died	Stayed	Left	Died
% of Initial Sample	49.8	47.8	2.7	64.6	35.2	0.24	42.3	56.4	1.3	60.3	39.0	0.7
Number of Chronic Conditions at Wave 1	0.4	0.4	1.0	0.3	0.2	2.0	0.4	0.4	0.7	0.3	0.3	0.0
Number of Functional Limitations at Wave 1	0.9	1.1	0.8	0.4	0.5	0	1.1	1.1	0.6	0.5	0.5	0.7
Number of Individuals	333	319	16	266	145	1	376	475	11	247	155	3
Number of Observations	1,451	667	35	1,187	303	4	1,770	872	32	1,204	341	11
	United Kingdom						United States					
	Lower-Educated			Higher-Educated			Lower-Educated			Higher-Educated		
	Stayed	Left	Died	Stayed	Left	Died	Stayed	Left	Died	Stayed	Left	Died
% of Initial Sample	46.9	45.0	8.1	67.6	27.1	5.3	68.7	22.0	9.3	77.3	18.0	4.7
Number of Chronic Conditions at Wave 1	0.4	0.5	0.8	0.3	0.4	0.4	0.8	0.7	1.5	0.5	0.5	1.1
Number of Functional Limitations at Wave 1	1.6	1.8	3.6	0.7	0.9	1.4	2.4	2.2	3.6	1.0	0.9	2.3
Number of Individuals	1,855	1,244	272	596	178	43	2,987	493	356	1,584	252	91
Number of Observations	12,240	2,946	678	3,980	483	122	14,440	1,415	726	7,759	702	198

*Notes:* Data are from SHARE (Sweden and Netherlands), ELSA (United Kingdom), and HRS (United States). Stayed = observed until the last panel wave. Left = left the panel before the last wave but did not die. Died = died before the last panel wave.

The data in Table 5 show that the lower-educated were more likely to die in each of the four countries, although such occurrences were rare in Sweden and the Netherlands. In line with previous evidence, this finding suggests that selective mortality leads to positive selection effects with regard to health among the lower-educated who remained under observation. This selection is further corroborated by the findings on health at initial observation, showing that those who later died reported more chronic conditions and functional limitations. Given that positive selection effects related to healthy survivors pertained more strongly to the United States and the United Kingdom than to Sweden and the Netherlands, these findings suggest that my estimates on better health among lower-educated people in Sweden and the Netherlands can be considered conservative.

Table 5 also shows that dropout for reasons other than death was more prevalent and more stratified by education in Sweden, the Netherlands, and the United Kingdom than in the United States. However, these differences in panel attrition were unrelated to the number of chronic conditions upon initial observation. Regarding functional limitations, lower-educated dropouts in Sweden and the United Kingdom (but not the Netherlands and the United States) reported worse health at initial observation.

Although the findings from Table 5 increase confidence in the main comparative conclusions, it is still possible that *changes* in health were related to different types of attrition. If Swedes who left the panel experienced steeper health declines before dropping out, for example, this might still bias the comparative findings even if initial levels of health were unrelated to panel attrition.

To correct for this potential source of bias, I reestimated the models using inverse probability weighting (IPW). To calculate the weights, I estimated a series of logistic regressions for participating in wave *t* as a function of explanatory variables (**X**_*t* – 1_) in a previous wave *t* – 1, conditional on having participated in wave *t* – 1 (Tchetgen et al. 2012; Wooldridge 2002). The IPWs for year *t* are then obtained as the inverse of the product of the predicted probabilities of staying in the panel (${\widehat{p}}_t$) until year *t* for *T* total years.

$$ {IPW}_t=\frac{1}{\prod_1^T{\widehat{p}}_t\left({\mathbf{X}}_{t-1}\right)}. $$

The main advantage of this approach is that the dynamics in the explanatory variables are used to predict survival. The variables included in the model to calculate IPWs were both health measures, age, cohort, education, gender, country, and interactions between each of these variables measured at *t* – 1. The approach gives higher influence to those individuals with a high probability of dropping out of the survey and lower influence to individuals with (health) characteristics leading to a lower probability of dropping out.

Figures S5 and S6 in Online Resource 1 show the results with IPWs and without IPWs. The unweighted results are presented in color, and the results after weighting are presented in gray. Results remained almost unchanged after using IPWs. The estimated number of functional limitations among lower-educated Swedes was slightly higher in the weighted model for the youngest cohort, but these small differences did not affect the substantive conclusions. Overall, the results obtained from IPW were in line with those reported by other studies on health over the life curse that used this method to correct for attrition. Although attrition was selective, IPW barely influenced the results (e.g., Baeten et al. 2013).

Note that these analyses did not account for a potential “healthy participant” effect (Morgan and Winship 2007). It was not possible to determine whether healthier individuals were more likely to participate in the surveys because the data producers provided no information about the extent to which nonresponse depended on health. If respondents in poorer health are less likely to participate in a telephone survey than in a face-to-face interview, this effect could explain part of the differences found between the United States and the three European countries. However, cross-national differences in line with my expectations emerged not only between the United States and the other countries but also between the three European countries, where data were collected in a similar way.

## Discussion

According to the cumulative (dis)advantage hypothesis, education reproduces and magnifies differences in health-related resources over the life course, producing growing education gaps in health from younger age to older age (Ross and Wu 1996). The present study focused on cross-national differences in this process, using comparative data on health trajectories in four countries. Sweden and the United States offer sharp contrasts with respect to inequality in health-related resources and risks over the life course (DiPrete 2002). The Netherlands and the UK can be placed between these poles, allowing for a nuanced test of comparative arguments.

I expected a gradient across the four countries (United States > United Kingdom > Netherlands > Sweden) in terms of the prevalence of health problems, the magnitude of health gaps between education groups, and the extent to which health gaps across education levels increase with age, as postulated by the cumulative (dis)advantage hypothesis*.* The results were largely in line with these expectations, revealing a clear cross-national gradient in chronic conditions and functional limitations and in the extent to which education shaped age trajectories in these measures of physical health.

First, across all ages and cohorts, physical health problems were more prevalent in older Americans among both the lower-educated and the higher-educated. Second, health gaps by education were largest in the United States, followed by the United Kingdom, the Netherlands, and Sweden. These differences in the magnitude of health differences between education groups indicate that the divergent pattern associated with the cumulative (dis)advantage hypothesis was most pronounced in the United States, less pronounced in the United Kingdom and the Netherlands, and least pronounced in Sweden. Although my data did not cover life course stages before the age of 50, my results for health differences between education groups at the start of the observation window suggest that these gaps opened up at younger ages.

Third, initial health gaps further widened across the observation window in all countries and in most of the cohorts studied within each country. These findings lend support to the cumulative (dis)advantage hypothesis for health trajectories in later life. As expected, the extent to which health inequality increased with age differed cross-nationally, indicating that divergence was smallest in Sweden, larger in the Netherlands and the United Kingdom, and largest in the United States.

In contrast to previous comparative studies of health inequality, these findings are based on a longitudinal design and comparable measures of education and health. Furthermore, unlike previous cross-sectional research, my findings are corroborated by attrition analyses that examined whether observed country differences in health inequality were biased by selective dropout. Additional sensitivity analyses showed that the cross-country gradient found in this study applied to men and women and was robust to racial and regional differences within the United States.

The longitudinal comparative data used in this study allowed me to account for cohort effects, an important requirement for the analysis of age trajectories in health (Lynch 2003). Although cohort effects should be interpreted with caution given the limited age overlaps between cohorts in my data, some notable patterns emerged from the analysis. In the United States, cohort effects were strong and consistent with the rising importance hypothesis, which posits that cumulative (dis)advantage has intensified in newer cohorts (Goesling 2007; Mirowsky and Ross 2008). This trend was mainly due to faster health declines among lower-educated Americans rather than slower declines among higher-educated Americans. I also found a pattern of rising importance for the Netherlands, where education groups increasingly drifted apart, resulting from accelerating health declines among the lower-educated combined with slowing health declines among the higher-educated. In the United Kingdom and Sweden, the data showed no indication of a rising importance of cumulative (dis)advantage in physical health trajectories.

The differences found across countries in the size of cohort effects are broadly in line with substantive arguments about the sociohistorical conditions surrounding successive birth cohorts. In the United States, the finding of increasing disparities in health by education has been attributed to a sharp and steady increase in economic inequality since the 1970s (Hayward et al. 2015; Mirowsky and Ross 2008). This rise of inequality was much larger in the United States than in other developed societies, including the United Kingdom and the Netherlands (Bambra and Beckfield 2012). In Sweden, inequality in health-related resources increased only slightly across the cohorts covered in the present study. These cohorts spent major stages of adult life during the golden age of the Swedish welfare state (Esping-Andersen 1999). Yet, although Sweden still ranks among the least unequal countries, income inequality has increased since the 1990s; the welfare state has been cut back; and social security, health care, and the labor market have become more liberal (Freeman et al. 2010). These changes suggest that health inequality could increase in newer cohorts, and this idea was supported in a recent study of cumulative (dis)advantage in Sweden (Leopold 2016).

Cross-country differences in cohort effects constitute another important source of contextual variation that should be addressed in future research. The data I used were not ideal to study these differences in detail, given the limitations regarding cohort range, observation periods, and overlaps between age and cohort. Internationally comparative panel data that cover more birth cohorts and follow them for a longer period of time are needed to analyze change in cross-country differences in health inequality.

Perhaps the most striking comparative result I found concerns cross-national differences in older people’s health found across all education groups. Most notably, *higher*-educated Americans were in similar or worse health than *lower*-educated people in the United Kingdom, the Netherlands, and Sweden. Confidence in this finding is strengthened by specific analytical benefits of the present study, which compared education groups that belonged to similar birth cohorts, were observed in similar historical periods, reported on the same health measures, and were not affected by compositional change related to differential rates of attrition. Moreover, this finding is consistent with studies by Avendano et al. (2009, 2010) reporting substantial disadvantages of wealthy and higher-educated Americans compared with Europeans in terms of life expectancy, chronic conditions, and functional limitations. Similarly, Banks et al. (2006) found that Americans belonging to the top third of the income distribution were comparable with those in the bottom third of the income distribution in England with regard to self-reported hypertension, diabetes, and a wide range of biological measures. Similar to the current study, these results were not driven by compositional effects with respect to race or region.

The present study adds to these findings by showing that the health disadvantage of lower- and higher-educated Americans compared with their Swedish, Dutch, and British counterparts did not level off with age or across cohorts. Explanations that have been advanced for these patterns are societal factors that expose both lower-educated and higher-educated Americans to more adverse living conditions and higher levels of stress. These societal factors include the obesity epidemic as well as social policy regarding infrastructure, employment protection, housing, family, and health care (Avendano and Kawachi 2014). In view of the current study’s results, a more detailed examination of the mechanisms underlying the prevalence of health problems among higher-educated Americans appears worthwhile.

On a more general level, this study’s findings add to knowledge on cross-national differences in health inequality between education groups. Previous comparative research was based largely on cross-sectional data (Avendano et al. 2010; Jürges 2010) and could not provide insight into life course–specific and cohort-specific processes that produce health gaps between education groups. My results have shown that these processes vary substantially across developed countries. An objective for future research is to study the mechanisms that underlie these differences. Addressing such mechanisms empirically is beyond the scope of this study, but the results support the general idea that education differences in health are profoundly shaped by contextual factors.

Understanding the role of these factors would require extending the comparative life course perspective to cover earlier stages in which health differences between education groups emerge. In this regard, I discussed a number of institutional factors that are relevant at different stages of life and in different domains of life and have the potential to intensify or to inhibit the increase in health inequality across lives and cohorts. Most, but not all, of the institutional factors are in line with cross-country gradient found in the analyses (United States > United Kingdom > Netherlands > Sweden). For example, some of the factors discussed suggest that the Dutch education system is more conducive to the growth of inequality over the life course than education systems in Sweden, the United Kingdom, and the United States. My findings on relatively small health differences between education groups in the Netherlands suggest that if these factors operate in the expected direction, they are less influential than countervailing factors. Future research is needed to understand the role of particular contextual factors in cross-country differences in health inequality over the life course.

Because addressing all these explanations simultaneously is difficult, future research should also examine key factors in detail. For example, the institutional factors that I outlined suggest country differences in stress and economic hardship, which may harm health directly or indirectly by triggering unhealthy behaviors. From a comparative point of view, the adverse effects of critical life events, such as union dissolution or job loss, could be larger in liberal economies (such as the United States and the United Kingdom) and smaller in protective welfare states (such as Sweden and the Netherlands).

Another important question for research and policy concerns country differences in terms of selection into education based on social background and health in childhood. Numerous studies have shown that parental education as well as early-life health and economic hardship predict educational attainment and partly explain the relationship between education and adult health (Behrman et al. 2011; Duke and Macmillan 2016; Lynch and Hippel 2015; Ross and Mirowsky 2011). In the absence of comparative studies, the role of institutional factors in moderating health selection into education remains unexplored. Certain features of education systems, such as an early tracking age, appear likely to tighten the link between childhood health and educational attainment. Studies that examine these connections would also clarify the extent to which cross-national differences in health inequality are due to social background and health selection into education.

Comparative research along these lines will improve our understanding of how to target health inequality by policy measures. The results of the present study suggest that the potential for reduction is substantial.

## Electronic supplementary material

ESM 1 (DOCX 406 kb)

## Footnotes

1 QIC is an equivalent to the Akaike information criterion (AIC) for Poisson-distributed generalized estimating equation (GEE) population-averaged models (Pan 2001) used in this study.

2 I tested for alternative specifications, such as hierarchical linear models and negative binomial models. The results were nearly identical to the GEE population-averaged model.

3 In ELSA, the birth years were 1952, 1949, 1946, 1943, 1940, and 1937.

4 Those born around 1950 are the 1951 cohorts in Sweden, the Netherlands, and the United States, and the 1949 cohort in the United Kingdom.
