# Supplementary material for: Education and Physical Health Trajectories in Later Life: A Comparative Study
Source: Demography. 2018 May 21;55(3):901–27. doi: 10.1007/s13524-018-0674-7 (PMC5992243; doi:10.1007/s13524-018-0674-7)
Supplement: Supplementary file 1 — (DOCX 406 kb) [file 13524_2018_674_MOESM1_ESM.docx]

**Online Resource 1**

Table S1. Multilevel Poisson Population-averaged Models

|  | Model 1 | | Model 2 | |
| --- | --- | --- | --- | --- |
|  | Number of Chronic Conditions | | Number of Functional Limitations | |
| Intercept | -0.52^***^ | (0.15) | 0.18 | (0.19) |
| Age | -0.0035 | (0.16) | -0.084 | (0.19) |
| Age squared | 0.00080 | (0.00) | 0.0012 | (0.00) |
| Cohort | -0.014 | (0.01) | -0.00087 | (0.02) |
| Education (ref. Lower) |  |  |  |  |
| Intermediate | -0.039 | (0.21) | -0.56^*^ | (0.27) |
| Higher | -0.74^**^ | (0.24) | -0.66^*^ | (0.28) |
| Country (ref. SE) |  |  |  |  |
| US | 0.65^***^ | (0.16) | 0.97^***^ | (0.20) |
| UK | 0.14 | (0.15) | 0.41^*^ | (0.19) |
| NL | 0.14 | (0.20) | 0.36 | (0.25) |
| Country x Age |  |  |  |  |
| US x Age | 0.25 | (0.17) | 0.31 | (0.21) |
| US x Age squared | -0.0024 | (0.00) | -0.0029 | (0.00) |
| UK x Age | 0.34^*^ | (0.16) | 0.020 | (0.20) |
| UK x Age squared | -0.0032^*^ | (0.00) | -0.00035 | (0.00) |
| NL x Age | -0.095 | (0.23) | -0.091 | (0.29) |
| NL x Age squared | 0.00054 | (0.00) | 0.00072 | (0.00) |
| Age x Education |  |  |  |  |
| Age x Intermediate | -0.019 | (0.03) | -0.024 | (0.03) |
| Age x Higher | -0.028 | (0.03) | -0.0068 | (0.03) |
| Age x Cohort | -0.0037 | (0.00) | -0.0047 | (0.00) |
| Cohort x Education |  |  |  |  |
| Cohort x Intermediate | -0.0092 | (0.02) | 0.032 | (0.03) |
| Cohort x Higher | 0.030 | (0.03) | -0.016 | (0.03) |
| Age x Cohort x Education |  |  |  |  |
| Age x Cohort x Intermediate | 0.00086 | (0.00) | 0.0042 | (0.00) |
| Age x Cohort x Higher | 0.0024 | (0.00) | 0.0041 | (0.00) |
| US x Education |  |  |  |  |
| US x Intermediate | -0.12 | (0.26) | 0.33 | (0.31) |
| US x Higher | 0.24 | (0.29) | -0.18 | (0.34) |
| UK x Education |  |  |  |  |
| UK x Intermediate | -0.081 | (0.23) | 0.32 | (0.29) |
| UK x Higher | 0.43 | (0.27) | -0.40 | (0.32) |
| NL x Education |  |  |  |  |
| NL x Intermediate | -0.31 | (0.31) | 0.097 | (0.40) |
| NL x Higher | -0.25 | (0.33) | -0.66 | (0.41) |
| US x Education x Age |  |  |  |  |
| US x Intermediate x Age | 0.016 | (0.03) | 0.018 | (0.03) |
| US x Higher x Age | 0.025 | (0.03) | 0.0033 | (0.04) |
| UK x Education x Age |  |  |  |  |
| UK x Intermediate x Age | 0.028 | (0.03) | 0.016 | (0.03) |
| UK x Higher x Age | 0.016 | (0.03) | 0.015 | (0.04) |
| NL x Education x Age |  |  |  |  |
| NL x Intermediate x Age | 0.0017 | (0.04) | 0.021 | (0.04) |
| NL x Higher x Age | -0.0041 | (0.04) | 0.027 | (0.05) |
| US x Cohort | -0.016 | (0.02) | -0.029 | (0.02) |
| UK x Cohort | -0.012 | (0.02) | 0.016 | (0.02) |
| NL x Cohort | -0.018 | (0.02) | -0.039 | (0.02) |
| US x Age x Cohort | 0.0058 | (0.00) | 0.0069 | (0.00) |
| UK x Age x Cohort | 0.0072^*^ | (0.00) | 0.0026 | (0.00) |
| NL x Age x Cohort | 0.0021 | (0.00) | 0.00014 | (0.00) |
| US x Education x Cohort |  |  |  |  |
| US x Intermediate x Cohort | 0.013 | (0.03) | -0.031 | (0.03) |
| US x Higher x Cohort | -0.021 | (0.03) | 0.026 | (0.04) |
|  | *continued on the next page* | | | |
|  | *TABLE S1 continued* | | | |
|  |  |  |  |  |
| UK x Education x Cohort |  |  |  |  |
| UK x Intermediate x Cohort | 0.0046 | (0.02) | -0.048 | (0.03) |
| UK x Higher x Cohort | -0.031 | (0.03) | 0.051 | (0.04) |
| NL x Education x Cohort |  |  |  |  |
| NL x Intermediate x Cohort | 0.019 | (0.04) | -0.023 | (0.04) |
| NL x Higher x Cohort | 0.039 | (0.04) | 0.089^*^ | (0.04) |
| US x Education x Cohort x Age |  |  |  |  |
| US x Intermediate x Cohort x Age | -0.00050 | (0.00) | -0.0027 | (0.00) |
| US x Higher x Cohort x Age | -0.0017 | (0.00) | -0.0020 | (0.00) |
| UK x Education x Cohort x Age |  |  |  |  |
| UK x Intermediate x Cohort x Age | -0.0017 | (0.00) | -0.0029 | (0.00) |
| UK x Higher x Cohort x Age | -0.0011 | (0.00) | -0.0048 | (0.00) |
| NL x Education x Cohort x Age |  |  |  |  |
| NL x Inter. x Cohort x Age | -0.0010 | (0.00) | -0.0037 | (0.00) |
| NL x Higher x Cohort x Age | -0.0014 | (0.00) | -0.0044 | (0.00) |
| *N* (observations) | 71,154 | | 71,073 | |

Note: Data are from SHARE (SE and NL), ELSA (UK), and HRS (US). Each of the data sets were weighted with individual cross-sectional weights for the wave of the initial observation; Standard errors in parentheses. *** p < 0.001, ** p < 0.01, * p < 0.05.

Figure S1. Predicted Aging Vectors of Chronic Conditions by Education, Gender, Race, and Country

Note: Data are from SHARE (SE and NL), ELSA (UK), and HRS (US).
Predictions shown in the left-hand column are based on Model 1, Table S1. Predictions in the middle and in the right-hand column are based on equivalent models estimated separately for men and women and for black people (black lines) and white people (grey lines) in the US.

Solid lines = Higher education. Dashed lines = Lower education.

Figure S2. Predicted Aging Vectors of Functional Limitations by Education, Gender, Race, and Country

Note: Data are from SHARE (SE and NL), ELSA (UK), and HRS (US).
Predictions shown in the left-hand column are based on Model 2, Table S1. Predictions in the middle and in the right-hand column are based on equivalent models estimated separately for men and women and for black people (black lines) and white people (grey lines) in the US.

Solid lines = Higher education. Dashed lines = Lower education.

Figure S3. Predicted Aging Vectors of Chronic Conditions by Education, Country, and Region within the US

Note: Data are from SHARE (SE and NL), ELSA (UK), and HRS (US). Predictions for Sweden, the Netherlands, the UK, and the US (all regions) are based on Model 1, Table S1. Predictions for regions are based on equivalent models estimated separately for each of the four regions.

Solid lines = Higher education. Dashed lines = Lower education.

Figure S4. Predicted Aging Vectors of Functional Limitations by Education, Country, and Region within the US

Note: Data are from SHARE (SE and NL), ELSA (UK), and HRS (US). Predictions for Sweden, the Netherlands, the UK, and the US (all regions) are based on Model 2, Table S1. Predictions for regions are based on equivalent models estimated separately for each of the four regions.

Solid lines = Higher education. Dashed lines = Lower education.

Figure S5. Predicted Aging Vectors of Chronic Conditions with and without Inverse Probability Weights

Note: Data are from SHARE (SE and NL), ELSA (UK), and HRS (US). Predictions in color are based on Model 1, Table S1. Predictions in grey are based on the same model using inverse probability weights.

Solid lines = Higher education. Dashed lines = Lower education.

Figure S6. Predicted Aging Vectors of Functional Limitations with and without Inverse Probability Weights

Note: Data are from SHARE (SE and NL), ELSA (UK), and HRS (US). Predictions in color are based on Model 2, Table S1. Predictions in grey are based on the same model using inverse probability weights.

Solid lines = Higher education. Dashed lines = Lower education.
